# Long Period Gratings in Random Hole Optical Fibers for Refractive Index Sensing

**DOI:** 10.3390/s110201558

**Published:** 2011-01-27

**Authors:** Ke Wang, Gary Pickrell

**Affiliations:** Center for Photonics Technology, Department of Materials Science and Engineering, Virginia Tech, Blacksburg, VA 24061, USA; E-Mail: pickrell@vt.edu

**Keywords:** long period grating, random hole optical fiber, point-by-point, refractive index sensing, **PACS**42.81.Pa, 42.81.Bm

## Abstract

We have demonstrated the fabrication of long period gratings in random hole optical fibers. The long period gratings are fabricated by a point-by-point technique using a CO_2_ laser. The gratings with a periodicity of 450 μm are fabricated and a maximum coupling efficiency of −9.81 dB has been achieved. Sensing of different refractive indices in the surrounding mediums is demonstrated by applying standard liquids with refractive indices from 1.400 to 1.440 to the long period grating.

## 1. Introduction

Random hole optical fibers (RHOFs) [1,2], being a new class of photonic crystal fibers (PCFs) [3,4] with their in-vivo generation of thousands of holes randomly distributed in the cladding while being drawn from a glass preform, have significantly reduced the fabrication difficulties compared with ordered-hole PCFs. Their counterparts, the ordered-hole PCFs, are commonly made by stacking tubes in a strict order and drawing the fiber from the tube array preform. During the fabrication of the ordered-hole PCFs, especially for the fibers guided by the photonic bandgap effect, any tiny disorder may result in serious degradation of the light guiding ability. Thus, because of their random nature of the hole arrangement in the cladding, RHOFs do not suffer the same penalty from the strict ordering requirements in the ordered-hole PCFs, significantly reducing the fabrication difficulties. With the unique advantages of the random hole structure in the cladding, RHOFs have shown great potential for a variety of sensing applications including gas sensing applications [5] and nuclear applications [6].

**Figure 1.**
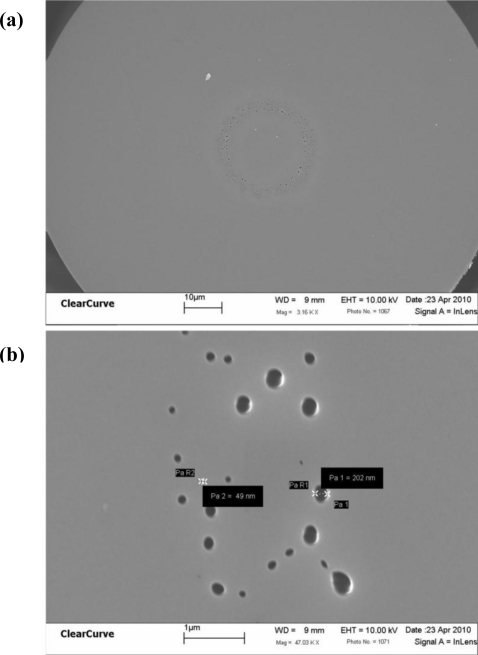
**(a)** An SEM micrograph of a random hole optical fiber. **(b)** Magnified random holes.

Unlike the critical requirements for photonic bandgap fibers, the random holes, ranging from nanometers up to micrometers in diameter, serve to reduce the effective refractive index of the cladding. As a result, light is guided in the core region, where the refractive index is higher than that of the cladding, by an index-guiding mechanism. An SEM micrograph of a piece single-mode RHOF endface is shown in Figure 1(a) and the magnified random holes are shown in Figure 1(b). From Figure 1, a large number of holes can be seen randomly distributed in the cladding. Like ordered-hole PCFs, the RHOF can be designed into excellent sensors for gas, chemical and biological sensing by introducing these sensing molecules into the random holes [5]. Especially when the RHOF is written with a long period grating (LPG), this proposed device is expected to have a higher sensitivity than an LPG written in regular fibers because it has a larger interaction area inside the random holes with the sensing medium. Moreover, previous studies have also shown that the RHOFs have very low bending loss compared with regular telecommunication fibers [7], and thus RHOF-based sensors can be developed for bend-sensitive applications with the requirement of very small bending radius.

On the other hand, the LPGs, whose periodicities ranging from 100 to 1,000 μm, have been extensively studied and utilized as sensing devices [8–11]. The fabrication of LPGs in telecommunication fibers has utilized various techniques such as the UV laser, the CO_2_ laser [12], the electric arc [13] and the mechanical method [14]. The fabrication of LPGs in RHOFs has also been accomplished by utilizing the electric arc from a fusion splicer [15]. However, writing LPGs with a CO_2_ laser is simple and cost effective compared with the UV-writing technique such as the high cost of the UV laser, optical components, and the complexity of the mechanical setup. In addition, the CO_2_ laser can be controlled more precisely in terms of pulse energy and duration, thus this technique can fabricate more consistent LPGs than using the electric arc technique. By carefully selecting the optimum fabrication conditions such as the laser power and lens focusing, the CO_2_ laser beam can slightly modify the fiber properties through the following three main mechanisms [16]: (i) residual stress relaxation; (ii) glass structure changes and (iii) physical deformation. As a result, the material refractive index is changed. Among the three mechanisms, glass structure changes and physical deformation in the fiber change the glass volume, densification and fiber core/cladding dimensions, and thus leading to a change in the effective refractive index too. This operation can be made to the fiber at a point-by-point manner until the fundamental core mode is coupled into strong cladding modes by the LPG. Indeed, the fiber experiences periodic deformations produced by the CO_2_ laser. The resonant wavelength λ satisfies the following equation [17]:

(1) $$\lambda =\left(n_{\mathrm{core}}-n_{\mathrm{cladding}}^{m}\right)\Lambda$$

where n_core_ is the effective refractive index of the core; n^m^_cladding_ is the effective refractive index of m^th^-order resonant wavelength in the cladding; and Λ is the periodicity of the LPG.

The LPGs in the RHOF can be used to sense changes in the refractive index in the surrounding medium and therefore have significant potential to be developed into gas, chemical and biological sensors because of the sensitivity to the external refractive index.

In this report, LPGs are written in the RHOF by utilizing a CO_2_ laser. To the authors’ knowledge, this is the first time that LPGs are experimentally demonstrated in the RHOF using a point-by-pint technique with a CO_2_ laser. Among these resultant LPGs, a maximum coupling efficiency of −9.81 dB has been achieved. In order to demonstrate the sensing potentials of the LPGs, wavelength response to the external refractive index has been also studied by immerging the LPG into several standard refractive index liquids.

## 2. Experiment

**Figure 2.**
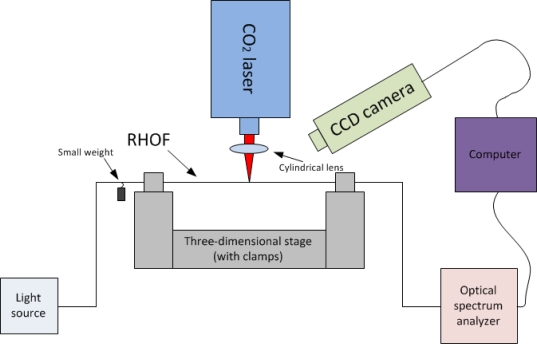
An illustration of the fabrication setup of the LPG written in the RHOF using a CO_2_ laser.

The fabrication setup was similar to what is previously reported for LPGs in regular telecommunication fibers [10,12,18,19]. As illustrated in Figure 2, a 40-cm-long single-mode RHOF was fusion spliced onto regular Corning SMF-28 telecommunication fibers. Then the whole fiber was connected with a broad-band light source and the optical transmission was monitored by an optical spectrum analyzer. The fiber was fixed on a three-dimensional stage (Thorlabs PT3/M, resolution 2 μm) through two fiber clamps (Thorlabs T711-250). A 3-g weight was hung on the fiber in order to maintain a constant stress in the fiber while the grating was being written. The small weight is important because the length is slightly increased when the fiber is melted by the laser. A CO_2_ laser (Synrad 48-2, wavelength 10.6 μm) was focused on to the RHOF through a cylindrical lens, whose focal length was 10 cm. The laser illumination time was precisely controlled by a computer program. The fabrication process was monitored by a CCD camera through an angle of 45°.

**Figure 3.**
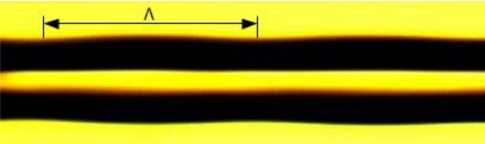
An optical micrograph of the deformation in the RHOF by the illumination of a CO_2_ laser. Such periodic deformations form the LPG, where Λ denotes the grating periodicity.

During the fabrication process, the laser power and illumination time were manually selected by virtually observing the thermal effect on the fiber, where only slight deformation induced by the heat from the laser pulses was observed through the CCD camera. The laser should induce sufficient change in refractive index but should not seriously weaken the fiber’s mechanical strength. Under the focusing by a 10-cm cylindrical lens, it was found that a suitable range was at 7% of the total power (25 W) and an illumination time of 3 s, such that LPGs can be successfully fabricated while maintaining sufficient their mechanical strength. Under the laser illumination at such conditions, the fiber was slightly melted, as seen from Figure 3. When it solidified, due to the glass structure changes and physical deformation to the core and the cladding, a slight changein the effective refractive index was achieved. After the deformation, the fiber was moved to the next point (one periodicity) by manually tuning the stage. Thus, the LPG was fabricated by a point-by-point technique, until strong cladding mode couplings were observed in the optical spectrum analyzer.

**Figure 4.**
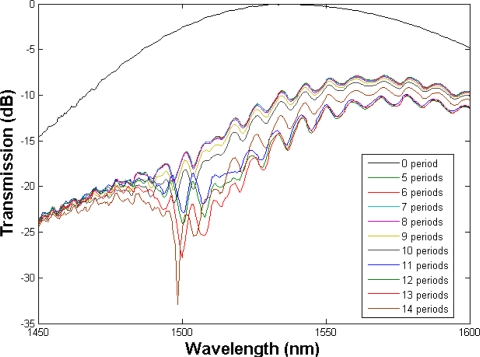
The transmission spectrum of an LPG in the RHOF with Λ = 450 µm.

In the experiment, the cladding mode coupling was normally observed after a few periods and started to reach the maximum coupling efficiency very quickly. For example, a typical grating transmission spectrum with a periodicity of 450 μm, is plotted. As it can be seen from Figure 4, the LPG reaches its maximum coupling efficiency of −9.81 dB at the wavelength 1,498.5 nm with 14 periods. After reaching the maximum coupling efficiency, the transmission dips were saturated, with no further changes observed even with more periods of laser inscriptions.

**Figure 5.**
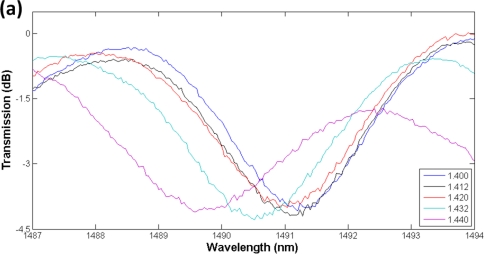

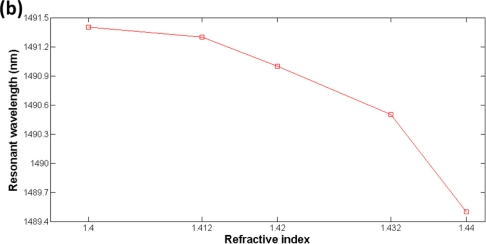
Resonant wavelength. **(a)** spectrums and **(b)** dips shift in response to the increase of refractive indices.

In order to investigate the feasibility for refractive index sensing, the LPG was immerged into standard refractive index liquids with refractive indices 1.400, 1.412, 1.420, 1.432 and 1.440, respectively. As shown in Figure 5, the LPG resonant wavelength shifts to lower wavelengths in response to the increase of the refractive indices in the surrounding mediums. This phenomenon is expected from (1) because n_cladding_^m^ is increased by larger refractive indices of the surrounding mediums thus leads to shorter resonant wavelengths.

## 3. Conclusion

In conclusion, we have demonstrated the fabrication of LPGs in RHOFs by a point-by-point technique using a CO_2_ laser. A maximum coupling efficiency of −9.81 dB has been achieved. Their sensing responses to the external refractive indices are also studied. These devices have great potentials to be developed into gas, chemical and biological sensors.

## References

[1] Kominsky D., Pickrell G., Stolen R. (2003). Generation of random-hole optical fiber. Opt. Lett.

[2] Pickrell G., Kominsky D., Stolen R., Ellis F., Kim J., Safaai-Jazi A., Wang A.B. (2004). Microstructural analysis of random hole optical fibers. IEEE Photonic. Tech. L.

[3] Knight J.C., Birks T.A., Russell P.S., Atkin D.M. (1996). All-silica single-mode optical fiber with photonic crystal cladding. Opt. Lett.

[4] Russell P. (2003). Photonic crystal fibers. Science.

[5] Pickrell G., Peng W., Wang A. (2004). Random-hole optical fiber evanescent-wave gas sensing. Opt. Lett.

[6] Alfeeli B., Pickrell G., Garland M.A., Wang A.B. (2007). Behavior of random hole optical fibers under gamma ray irradiation and its potential use in radiation sensing applications. Sensors.

[7] Pickrell G., Ma C., Wang A. (2008). Bending-induced optical loss in random-hole optical fibers. Opt. Lett.

[8] Kapoor A., Sharma E.K. (2009). Long period grating refractive-index sensor: Optimal design for single wavelength interrogation. Appl. Optics.

[9] Jiang M., Zhang A.P., Wang Y.C., Tam H.Y., He S.L. (2009). Fabrication of a compact reflective long-period grating sensor with a cladding-mode-selective fiber end-face mirror. Opt. Express.

[10] Wang K., Klimov D., Kolber Z. (2009). Seawater pH sensor based on the long period grating in a single-mode-multimode-single-mode structure. Opt. Eng.

[11] Zhu T., Rao Y.J., Wang J.L., Song Y. (2007). A highly sensitive fiber-optic refractive index sensor based on an edge-written long-period fiber grating. IEEE Photonic. Tech. L.

[12] Davis D.D., Gaylord T.K., Glytsis E.N., Kosinski S.G., Mettler S.C., Vengsarkar A.M. (1998). Long-period fibre grating fabrication with focused CO2 laser pulses. Electron. Lett.

[13] Hwang I.K., Yun S.H., Kim B.Y. (1999). Long-period fiber gratings based on periodic microbends. Opt. Lett.

[14] Rego G. (2008). Long-period fiber gratings mechanically induced by winding a string around a fiber/grooved tube set. Microw Opt. Techn. Let.

[15] Korwin-Pawlowski M.L., Pickrell G., Mikulic P., Du H.H., Fudouzi H. (2007). Long Period Gratings on Random Hole Optical Fibers and Microstructured Disordered Fibers.

[16] Wang Y.P. (2010). Review of long period fiber gratings written by CO2 laser. J. Appl. Phys.

[17] Kashyap R. (2010). Fiber Bragg Gratings.

[18] Wang K., Klimov D., Kolber Z. Long Period Grating-Based Fiber-Optic PH Sensor for Ocean Monitoring.

[19] Wang K., Klimov D., Kolber Z. Long Period Grating-Based Ocean pH Sensor in an SMS Fiber.

